# Development of Cellulose Nanofiber—SnO_2_ Supported Nanocomposite as Substrate Materials for High-Performance Lithium-Ion Batteries

**DOI:** 10.3390/nano13061080

**Published:** 2023-03-16

**Authors:** Quang Nhat Tran, Hyung Wook Choi

**Affiliations:** 1 Department of Chemical and Biological Engineering, Gachon University, Seongnam 13120, Gyeonggi-Do, Republic of Korea; 2 Department of Electrical Engineering, Gachon University, Seongnam 13120, Gyeonggi-Do, Republic of Korea

**Keywords:** lithium-ion batteries, cellulose nanofiber, SnO_2_, tin oxides, nanocomposite, energy storage

## Abstract

The large volumetric expansion of conversion-type anode materials (CTAMs) based on transition-metal oxides is still a big challenge for lithium-ion batteries (LIBs). An obtained nanocomposite was established by tin oxide (SnO_2_) nanoparticles embedding in cellulose nanofiber (SnO_2_-CNFi), and was developed in our research to take advantage of the tin oxide’s high theoretical specific capacity and the cellulose nanofiber support structure to restrain the volume expansion of transition-metal oxides. The nanocomposite utilized as electrodes in lithium-ion batteries not only inhibited volume growth but also contributed to enhancing electrode electrochemical performance, resulting in the good capacity maintainability of the LIBs electrode during the cycling process. The SnO_2_-CNFi nanocomposite electrode delivered a specific discharge capacity of 619 mAh g^−1^ after 200 working cycles at the current rate of 100 mA g^−1^. Moreover, the coulombic efficiency remained above 99% after 200 cycles showing the good stability of the electrode, and promising potential for commercial activity of nanocomposites electrode.

## 1. Introduction

The widespread success of lithium-ion batteries (LIBs) in areas such as electronic devices, electric vehicles, and power grids leads to increasing demand in the research and development of high-energy density and capacity electrode materials for next-generation LIBs. Among various candidates, transition-metal compounds based on the conversion reaction mechanism have attracted great interest because of their high theoretical specific capacities. Moreover, the conversion-type anode materials (CTAMs) based on the wide range of transition-metal oxides show great potential in expanding the material selection for high-performance LIBs [1,2,3,4,5]. Moreover, the natural forms of many CTAMs (e.g., Fe_3_O_4_, Fe_2_O_3_, FeS_2_, MnO_2,_ and SnO_2_) can help to reduce production costs in comparison with that of alloying-type anode materials. In addition, the reaction potentials of CTAMs could be adjusted based on bond strength between transition-metal cations and the anionic species, and the following ensures better battery safety by avoiding the lithium dendrite formation problem. Due to their higher specificity and safety compared to intercalation-type materials, as well as lower manufacturing costs compared to alloying-type materials, CTAMs are more promising for next-generation LIBs [1,6,7].

Among various conversion-type anode materials, the low cost, environmental benignity, and high abundance of Tin (Sn), tin oxide (SnO_2_) is considered one of the most preferred candidates. In addition, Sn-based compounds (such as oxides) can alloy with Li^+^ and undergo a conversion reaction, which exhibits higher electrochemical performance and capacity [8,9,10,11,12,13,14,15]. However, similar to other CTAMs, SnO_2_ still faces significant challenges, and relatively large volume expansion (<300%) during the insertion of Li^+^ into the Sn structure process (Sn + xLi^+^ + xe^-^ ↔ Li_x_Sn (0 ≤ x ≤ 4.4)), resulting in strong pulverization and loss of electrical contact between the active material and the carbon conductor. This leads to the result that the operating capacity of the SnO_2_-based anode is much lower than the theoretical capacity of SnO_2_ (790 mAh g^−1^) [12,16,17,18,19,20,21,22,23,24].

The development of nanostructures of the CTAMs, which have been obtained recently, not only improved electrochemical performance but also enhanced capacity and battery stability by standing up to the volume changes during cycling [1]. Moreover, the hybridization of CTAMs with various carbonaceous materials is another effective strategy for high-performance CTAMs in LIBs [1,25,26,27,28,29]. In addition, the good elasticity of carbonaceous materials will effectively adapt to volume change deformation during the insertion and extraction process of Li^+^, providing more advantages to the stability of the active materials during the cycling performance of LIBs [27,28,29]. There are three nanocomposite approaches based on the topology between carbonaceous materials and CTAMs, including coating carbonaceous materials on CTAMs, growing CTAMs on carbonaceous materials, and inserting CTAM nanoparticles into carbonaceous matrices [25,26,27,28,29]. Among them, the incorporation of CTAMs nanomaterials into these carbonaceous matrices could increase their lithium storage properties by virtue of their diverse functions and interdependent effects in nanocomposites. Furthermore, the advanced design of CTAMs nanoparticles insertion into carbonaceous matrix can also be easily attained through thermal annealing of inorganic–organic hybrid compounds. Moreover, the affluent chemistry of the organic ingredient in the predecessors can yield heteroatomic impurities to further improve the electrochemical activity by modulating the bandgap and/or changing the surface properties [20,23,24,30,31,32,33,34,35].

Cellulose nanofiber (CNFi), which can be derived from plants or produced by bacteria, is one of the most abundant green resources on Earth. CNFi has many attractive properties including low thermal expansion coefficient, high strength, high stiffness, easily modifiable surface, high crystallinity, naturally produced porous network, and good dispersibility in water, making CNFi an ideal carbonaceous matrix for constructing embedded CTAMs high-performance materials [36,37,38,39,40,41]. Furthermore, cellulose materials contain sodium carboxylate groups, which can dissociate the sodium ion into electrolytes, improve the formation of stable solid electrolyte interphase (SEI) layer, and enhance LIBs stability during cycling [42,43,44,45,46,47]. In addition, the low-cost, high-performance, and environmental-friendly alternative for the engineering requirement of cellulose nanofiber could contribute to reducing the production cost of materials for LIB applications.

In this study, we highlighted and developed a cellulose-based nanocomposite, which takes the advantages of CTAMs materials and cellulose nanofiber by thermally embedding SnO_2_ nanoparticles in cellulose nanofiber (SnO_2_-CNFi). The nanocomposite can further be used as electrode material in LIBs. The nanocomposite could effectively address the volume expansion of SnO_2_ and provide a highly conductive framework for enhanced rate capability. Moreover, the thermal treating process to embed SnO_2_ nanoparticles into the cellulose nanofiber could enhance the electrical conductivity of cellulose-based materials. Thus, the nanocomposite also exhibits excellent rate performance and good cycling stability as an anode material in LIBs. Its mass production can be achieved on large scale at a low cost for LIBs manufacturing.

## 2. Experiment Details

### 2.1. Chemicals and Reagents

The CNFi suspension obtained from SK Innovation Co. Ltd. (Daejeon, Korea) was used as a source of cellulose nanofiber for synthesizing the nanocomposite from SnO_2_ and CNFi. Tin (II) chloride dihydrate (SnCl_2_·2H_2_O) and sodium citrate dihydrate (C_6_H_5_Na_3_O_7_·2H_2_O) were purchased from Sigma-Aldrich reagent Co. Ltd. (St. Louis, MO, USA). Super-P amorphous carbon black (C, approximately 40 nm, 99.99%) was purchased from Alpha Aesar, Inc. (Ward Hill, MA, USA). Ethanol and deionized water were used throughout the synthesis of the nanocomposite.

### 2.2. Synthesis of SnO_2_-CNFi Nanocomposite

The SnO_2_-CNFi nanocomposite was prepared by a modified approach, as previously described [20,35]. Typically, 0.1128 g of SnCl_2_·2H_2_O and 0.2941 g C_6_H_5_Na_3_O_7_·2H_2_O were added into 40 mL ethanol–deionized (DI) water (1:1) solution. After being magnetically vigorously stirred for 1 h, the resulting solution was then transferred to a 100 mL stainless steel autoclave and the Cellulose Nanofiber (CNFi, 0.1 g) was added. The reaction was carried out at 180 °C for 8 h and was naturally cooled to room temperature. The obtained sample was collected by centrifugation, rinsed with DI water, and dried at 25 °C for 1 day. Then, the precursor was heat-treated at 500 °C for 2 h under nitrogen atmosphere with a temperature ramp of 5 °C min^−1^. The prepared nanocomposite was designated as the high-performance Li-storage material for LIBs. As for a comparison SnO_2_ material sample, the similar synthesis method under same condition was carried out without the presence of cellulose nanofiber.

### 2.3. Materials Characterization

Scanning electron microscopy (SEM, S-4700, Hitachi Ltd., Tokyo, Japan) and transmission electron microscopy (TEM, Tecnai, F30S-Twin, Hillsboro, OR, USA) images were taken to characterize the morphologies and structures of the sample nanocomposite, and elemental maps were obtained by energy dispersive X-ray analysis (EDX). X-ray diffraction (XRD) patterns were recorded over the 2θ range of 10–80° at a scanning rate of 1.0° min^−1^ on a diffractometer (Rigaku/Smartlab, Tokyo, Japan) with a Kβ filter for Cu radiation (40 kV, 30 mA X-Ray generator), provided by Smart Materials Research Center for IoT, at Gachon University. The content of SnO_2_ in the nanocomposite was determined using thermogravimetric analysis (TGA) with a temperature increase rate of 10 °C min^−1^ under atmospheric conditions and Brunauer–Emmett–Teller (BET) specific surface areas of SnO_2_-CNFi composites were determined by N_2_ adsorption at 77.3 K (Micromeritics, ASAP 2020). X-ray photoelectron spectroscopy (XPS, PHI 5000, Chigasaki, Japan) was introduced to determine the element content of the sample.

### 2.4. Electrochemical Performance Measurement

The SnO_2_-CNFi electrode (mass load 0.88 mg/cm^2^) was prepared by mixing 70% sample, 15% carbon black, and 15% polyvinylidene fluoride (PVDF) and dissolving into N-methyl pyrrolidinone (NMP) to form a slurry, which was then coated onto a copper foil (r = 0.6 cm) and dried overnight at 70 °C in a vacuum for 24 h. The CR2032-type coin cell (Rotech Inc., Gwangju, Korea) was assembled in a glove box filled with pure argon. Metallic lithium was used as a lithium reference counter electrode, about 50 μL of a solution consisting of 1 mol/L LiPF_6_ in ethylene carbonate (EC)/diethyl carbonate (DMC) mixture (1:1, by volume) were used as an electrolyte for each electrode, and polyethylene membrane was used as the separator. Galvanostatic discharge/charge experiments were performed over a potential range of 3~0.01 V vs. Li^+^/Li using a battery cycler (NanoCycler-01, NANOBASE, Geumcheon-gu, Seoul, Korea) system under a constant current density of 100 mA g^−1^ at room temperature. Subsequently, the rate performance tests were performed using various current densities in the range of 100–10,000 mA g^−1^. The cyclic voltammograms (CV) were tested from 0.01 to 3.0 V at a scan rate of 0.1 mV s^− 1^ battery-cycle tester (WBCS3000, WonAtech, Seocho-gu, Seoul, Korea). 100 kHz–100 MHz frequency range at an AC amplitude of 10 mV was used to conduct the electrochemical impedance spectra (EIS) test by ZIVE MP1 (WonATech, Seocho-gu, Seoul, Korea) analyzer.

## 3. Results and Discussion

### 3.1. Physical Properties of SnO_2_-CNFi Nanocomposite

**Figure 1:**
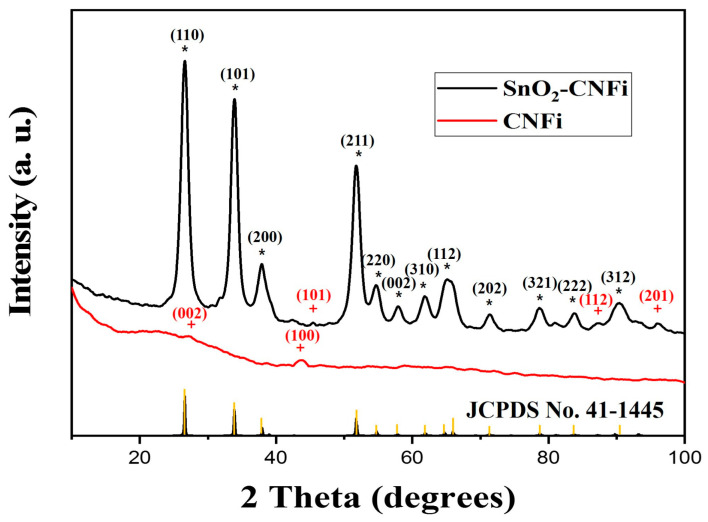
XRD profiles of CNFi and SnO_2_-CNFi. (*) for SnO_2_ peaks, and (+) for C peaks.

Figure 1 displays the XRD profiles of both CNFi and SnO_2_-CNFi. As shown in Figure 1, for CNFi, there are only two diffraction peaks at around 27.5 and 43° corresponding to the (002) and (100) planes of CNFi, in good agreement with the data provided by SK Innovation Co. Ltd. Meanwhile, the characteristic peaks of SnO_2_-CNFi existed highest and sharp peaks at 2θ = 26.6 (110), 33.8 (101), 37.9 (200), and 51.8° (211), which represented the well matching with the planes of SnO_2_ phase (JCPDS no. 41-1445/ICCD card no. 01-077-0449). Moreover, it is noted that more low indexed diffraction peaks were obtained at 54.7, 58, 62, 65.2, 71.2, 78.7, 90, and 93.2° corresponding to the (220), (002), (310), (112), (202), (321), (222), and (312) planes of SnO_2_, respectively (JCPDS no. 41-1445/ICCD card no. 01-077-0449). This also indicates the presence of SnO_2_ in the SnO_2_-CNFi composite. Furthermore, the weak peak has been slightly shifted from 2θ = 43° (100) in CNFi to 45° (101) in the SnO_2_-CNFi XRD pattern and two small peaks existed at 84 (112) and 96° (201) where the peaks are assigned to carbon (Graphite) (ICCD card no. 01-083-6084). The appearance of carbon structure diffraction peaks and also the independent peaks of SnO_2_ indicated that the SnO_2_ nanoparticles had been well introduced into nanocomposite and the subsequent oxidization process accompanied the transformation of cellulose into the conductive carbonaceous matrix, which not only produced a porous network for embedding SnO_2_ nanoparticles but also enhance the conductivity of the final product.

**Figure 2:**
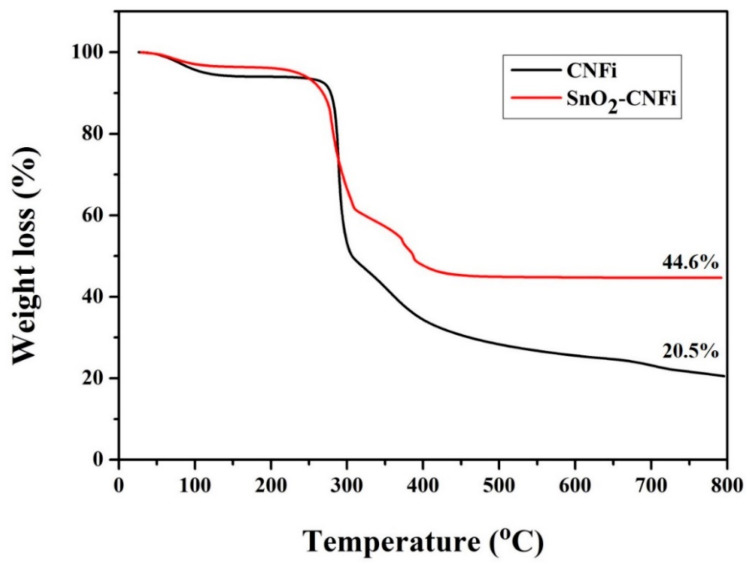
TGA curves of CNFi and SnO_2_-CNFi nanocomposite.

To further understand the composite of SnO_2_ in the nanocomposite, the thermogravimetric analysis of CNFi and SnO_2_-CNFi nanocomposite product were carried out and the results were shown in Figure 2. According to the TGA curves, the decomposition reaction of SnO_2_-CNFi started from 300 °C to 650 °C in the atmospheric conditions and the composite of SnO_2_ is about 24 wt%.

**Figure 3:**
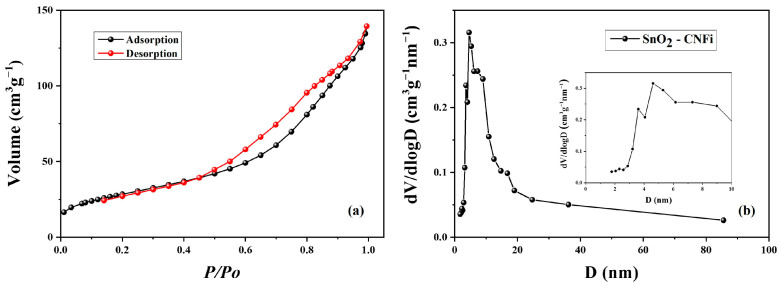
(**a**) Nitrogen adsorption–desorption isotherm and (**b**) BJH pore size distribution of the nanocomposite.

The porosities of SnO_2_-CNFi nanocomposite are evaluated by nitrogen isothermal adsorption and desorption measurements and the results were shown in Figure 3. The Brunauer–Emmett–Teller Specific surface areas (SSA) and pore volume (PV) of SnO_2_-CNFi were calculated to be 144.2 m^2^ g^−1^ and 0.208 cm^3^ g^−1^, respectively. According to the shape, the isotherm of SnO2-CNFi is allocated as type-IV characteristic with a H3-type hysteresis loop based on the small uptake in low range and absence of limiting adsorption at high relative pressure (P/P_0_) [48,49]. The obtained hysteresis slope classtifies the nanocomposite exhibit the presence of mesopores (2–50 nm) and have very likely micropores (<2 nm). Further more, the SSA of SnO_2_-CNFi (Figure 3a) is significantly higher than those of CNFi (96 m^2^ g^−1^) and based on the contribution of SnO_2_ nanoparticles porous, which will enhance the contact with electrolyte, provide more storage space for lithium ion and rise the electrochemical reactive activity. The pore size distribution, as shown in Figure 3b, reveals the hierarchical porous structure of SnO_2_-CNFi, with the pores, ranging between 2–20 nm and centered at 8 nm, containing both nanopores and mesopores. The mesopores structure will shorten the Li^+^ diffusion path and the appearance of nanopores acting as buffering spaces, which conduct a volume change in SnO_2_ instead of being destroyed in the lithiation/de-lithiation process, which can prevent the volume expansion of SnO_2_ and enhance the specific capacity of the electrode and cycling stability.

**Figure 4:**
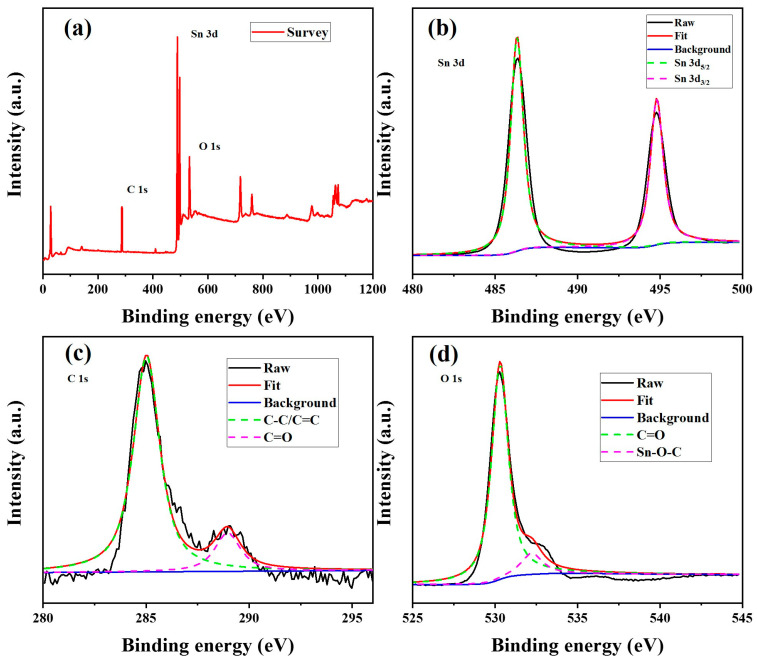
(**a**) XPS survey spectra of SnO_2_-CNFi and high-resolution XPS peaks of (**b**) Sn 3d, (**c**) C 1s and (**d**) O 1s.

The successful SnO_2_ nanoparticles attached to the carbonaceous material were analyzed by X-ray photoelectron spectroscopy (XPS). The chemical elements content and valence states were shown in Figure 4. The XPS spectrum of SnO_2_-CNFi nanocomposite (Figure 4a) verified the existence of C 1s, Sn 3d, and O 1s with the peaks placed at 287.27, 488.82, and 533.07 eV, respectively, which validate the presence of SnO2 and carbonaceous material in the nanocomposite. In particular, there are two peaks located at 486.38 eV and 494.78 eV, corresponding to peaks of Sn 3d_5/2_ and Sn 3d_3/2_ were observed in Sn 3d spectrum (Figure 4b). The XPS spectrums of C 1s and O 1s were displayed in Figure 4c,d with the corresponding peaks around 284.94, 288.6, 530.28, and 532.58 eV, related to the existence of C-C/C=C, C=O, O=C, and Sn-O-C bond, respectively, in the nanocomposite [13,19]. These results determined the successful embedding of SnO_2_ nanoparticles into the CNFi.

**Figure 5:**
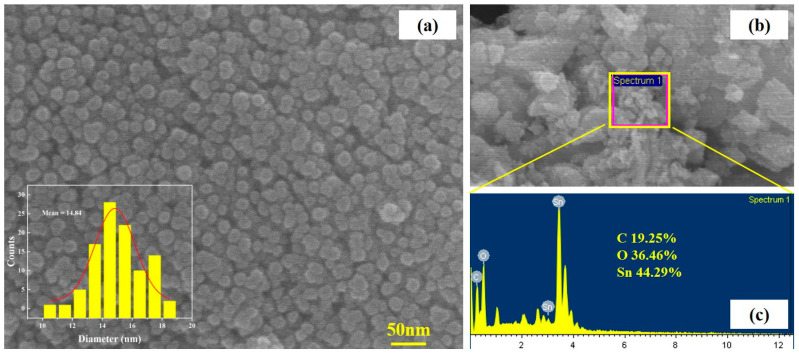
(**a**) SEM images and (**b**,**c**) EDX pattern of SnO_2_-CNFi nanocomposite.

**Figure 6:**
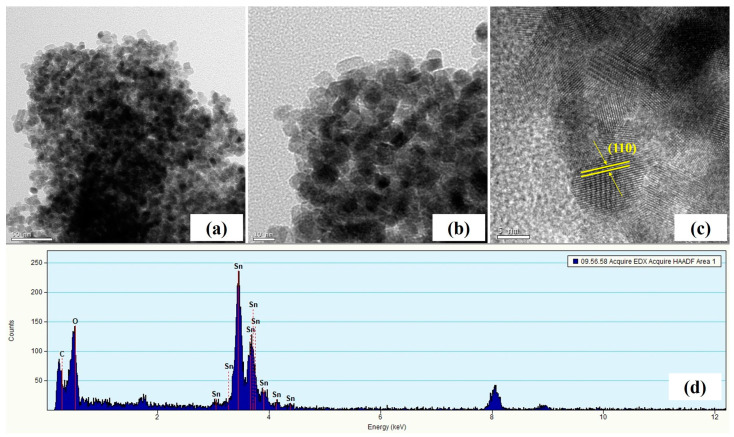
(**a**) Typical TEM and (**b**,**c**) HRTEM images, (**d**) EDX pattern of SnO_2_-CNFi nanocomposite.

At the same time, the surface and detailed morphologies of SnO_2_-CNFi nanocomposite were investigated by SEM and TEM and the results were shown in Figure 5 and Figure 6. From the SnO_2_-CNFi SEM image (Figure 5a), the nanocomposite exhibited a uniform surface morphology with spherical particles of SnO_2_. The insert showed the size distribution of SnO_2_ nanoparticles. The average size of SnO_2_ Nanoparticles is about 15 nm and has a narrow particle size distribution and good dispersion. Moreover, SnO_2_ nanoparticles also were uniformly distributed in the carbonaceous material and will help to prevent the aggregation of SnO_2_ nanoparticles during the cycling test [13,19,20,23,24,35]. Furthermore, the surface morphology of SnO_2_-CNFi after 50 cycles and EDX mapping were studied and performed in Figure 5b,c. The result (Figure 5b) clearly confirms that the morphology was still conserved after the cycling test and the composite structure still in a good shape, which proves the better combination of SnO_2_ and carbonaceous material. In addition, this means that the nano compound of SnO_2_ and CNFi has the potential to be used in the production of battery electrode materials. EDX mapping of SnO_2_-CNFi nanocomposite is shown in Figure 5c, three elements (Sn, O, C) are explored in the nanocomposite with the percentage contents 44.29%, 36.46%, and 19.25%, respectively, which again confirms the SnO_2_ nanoparticles were successfully attached to the carbonaceous material and consistently agrees with the XRD and XPS results.

To further confirm the clearly detailed morphologies and structure of SnO_2_-CNFi nanocomposite, TEM and high-resolution TEM (HRTEM) were carried out and the obtained images are displayed in Figure 6. TEM images of SnO_2_-CNFi (Figure 6a,b) present the uniform distribution of SnO_2_ nanoparticles in nanocomposite without forming large aggregation. It is evident that the particles are basically spherical in shape and exhibit an average particle size of 15 nm, proving that these results completely correspond to the obtained SEM images. Furthermore, the magnified HRTEM image (Figure 6c) confirms the presence of highly crystalline SnO_2_ nanoparticles with a lattice parameter is about 0.33 nm, corresponding to the (110) plane of the crystal structure of the nanocrystalline SnO_2_, which is consistent with the XRD results. Moreover, the amorphous carbon structure that appears in the gained results also confirms the successful embedding of nanoparticles into carbonaceous materials, resulting in the good form of the nanocomposite, which will effectively relax the drastic volume expansion of SnO_2_ nanoparticles during the charge–discharge process [17,19,20,24,35].

**Figure 7:**
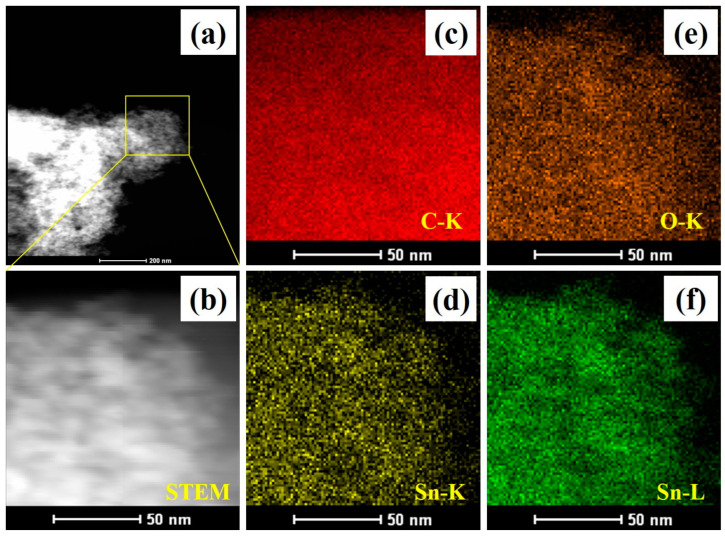
(**a**) TEM, (**b**) STEM, and full elemental mapping images (**c**) C, (**d**,**f**) Sn, and (**e**) O of SnO_2_-CNFi nanocomposite.

The successful embedding of SnO_2_ into the carbonaceous material was demonstrated by energy dispersive X-ray analysis (EDX) (Figure 6d). The EDX pattern of SnO_2_-CNFi clearly indicated the presence of tin (Sn), carbon (C), and oxygen (O). In addition, EDX elemental mapping of the SnO_2_-CNFi nanocomposite shown in Figure 7 is reliable to further confirm the successfully attached of SnO_2_ and the uniform distribution of SnO_2_ and CNFi. The results demonstrate three elements Sn, C, and O were found in the nanocomposite. With all the above results, it is verified that the SnO_2_-CNFi nanocomposite has been successfully synthesized as per our expectations. These embedding SnO_2_ nanoparticles in cellulose nanofiber-based carbonaceous materials efficiently expand the ability to prevent volume change, and agglomeration of SnO_2_ and improve the electrical performance of electrode materials.

### 3.2. Electrochemical Performance

**Figure 8:**
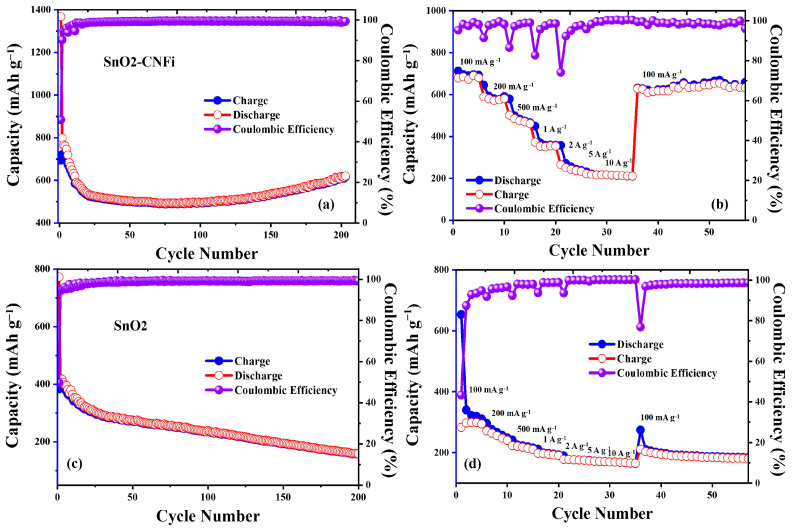
(**a**,**c**) Cycling performance and coulombic efficiency at 100 mA g^−1^ and (**b**,**d**) rate capabilities of SnO_2_-CNFi nanocomposite and bare SnO_2_ electrodes.

In order to investigate the electrochemical properties of the obtained nanocomposite for use as electrode materials in lithium-ion batteries, galvanostatic charge, and discharge tests were carried out with a potential range of 0.01–3.00 V at a constant current density of 100 mA g^−1^ was applied. The SnO_2_-CNFi nanocomposite electrode discharge, charge capacities, and cycling performance efficiency of 200 cycles are shown in Figure 8a. SnO_2_-CNFi nanocomposite exhibits initial discharge capacity and charge capacity of 1367.6, and 695.4 mAh g^−1^, respectively. However, the coulombic efficiency (CE) of nanocomposite only achieved 50.8%, which is the result of the Li ion embedded into mesopores of SnO_2_-CNFi nanocomposite during forming of the SEI film process. This would partially deplete more Li ions and lead to low coulombic efficiency [49,50]. Following, the electrode displays a significant decrease in capacity during the first 5 cycles and a rapid capacity decrease from 718.6 mAh g^−1^ to 503.2 mAh g^−1^ after 50 cycles. The capacity has no change, maintained for the next 50 cycles, and starts to increase gradually and reach a steady value of approximately 619 mAh g^−1^ in the subsequent 200 cycles. One phenomenon that can be easily seen is the capacity tends to increase faster after 150 cycles and achieved a capacity of 78.4% compared to the theoretical capacity of SnO_2_ and retained 45% of its inception capacity after 200 cycles. The phenomenon that capacity decreases first and then increases in cycling was the characteristic phenomenon of SnO_2_-based nanocomposite, which has been discussed in many previous reports [13,51]. The formation of Sn nanoparticles based on the pulverization of SnO_2_ during the lithiation process could be the reason causes the attenuation of capacity during the first 50 cycles. Moreover, the size of Sn nanoparticles also decreases during the charge–discharge process because of the electrochemical milling effect. The very small size of Sn particles could cause the reversible reaction (Sn → SnO_2_) in the SnO_2_-CNFi nanocomposite. However, the reversible reaction decreases with the increase in working cycles caused by the aggregation of Sn nanoparticles. Although typical initial capacity decreases due to the pulverization of SnO_2_ in the nanocomposite during the lithiation process and the loss of crystallinity of the nanosized SnO_2_ particles during the cyclability of nanocomposite electrode, the tolerances and flexibility of incorporated CNFi are better than the embedded metal oxide particles, making SnO_2_-CNFi nanocomposite anode easy to adjust to volume changes during lithiation and increasing the capacity after cycling [20,38,39,42,52]. In addition, the mesopores structure and large BET-specific surface (Figure 3) of SnO_2_-CNFi nanocomposite could work as a buffering structure against the aggregation and volume expansion and increase the capacity after cycling. Moreover, the CEs remain over 99% during the whole cycling process except the first cycle. These capacity residuals and excellent cycling stabilities establish a significant stable effective impact on the cyclability of nanocomposite. These results confirm that the CNFi and the SnO_2_-CNFi nanocomposite structures provide outstanding reversible capacity, minimize the volume expansion, enhance electrochemical performance, and result in stable cycling during the charge–discharge process.

The rate performances of SnO_2_-CNFi nanocomposite at different current rates from 100 mA g^−1^ to 10 A g^−1^ for every five successive cycles are shown in Figure 8b. The results show that SnO_2_-CNFi nanocomposite delivers specific average capacities of 697.7, 597.4, 500.3, 379.6, 273.5, 220.8, and 212.5 mAh g^−1^ at 0.1, 0.2, 0.5, 1, 2, 5, and 10 A g^−1^, respectively. Moreover, as the current rate returns to 100 mA g^−1^, the discharge capacity of SnO_2_-CNFi nanocomposite is recovered to 631.2 mAh g^−1^ after undergoing cycles at higher current densities, which is as high as 90.4 % of the initial value and even rapidly increase to 669.3 mAh g^−1^ after 20 cycles. The results indicate slow reaction kinetics of Li ions insertion/extraction in SnO_2_-CNFi nanocomposite.

Furthermore, the nanocomposite maintained a capacity of 231.5 mAh g^−1^ at a higher density of 5 A g^−1^, which shows a good rate performance in high current densities. Although relatively lower capacities are observed at a higher rate of 10 A g^−1^, the SnO_2_-CNFi nanocomposite electrode still harvests stable cycling capability with current rates below 5 A g^−1^.

Moreover, the coulombic efficiencies show a similar trend with a drop at the first cycle of every different current density. However, the CE did not show apparent change as the current density increased. The CEs return and remain above 99% with further cycling. These results again confirm SnO_2_-CNFi nanocomposite electrodes perform excellent stability and good rate-cycling performance of the electrode at various current densities.

Compared with SnO_2_-CNFi electrode, the galvanostatic charge–discharge tests and rate performance of the bare SnO_2_ electrode were investigated under the same condition to demonstrate the better electrochemical performance of SnO_2_-CNFi electrodes. The capacities, and cycling performance efficiency of 200 cycles of SnO_2_ electrodes were shown in Figure 8c. The discharge capacity of SnO_2_ electrodes was 772.65 mAh g^−1^ for the first cycle, which is much lower than the initial discharge capacity of SnO_2_-CNFi electrodes (1367.6 mAh g^−1^). However, as shown in previous works, the capacities of bare SnO_2_ electrodes gradually decrease over time and remained only at 158.67 mAh g^−1^ after 200 cycles, only a quarter of the capacity of SnO_2_-CNFi electrodes. Moreover, the capacities tend to decrease after cycling instead of tending to increase again after 50 cycles in comparison with SnO_2_-CNFi electrodes.

Following, the rate performance of bare SnO_2_ electrode for every five successive cycles delivers specific average capacities of 389.21, 268.86, 224.85, 199.14, 179, 171.44, and 166.93 mAh g^−1^ at 0.1, 0.2, 0.5, 1, 2, 5, and 10 A g^−1^, respectively, as displayed in Figure 8d. These results are markedly lower than the average capacities of SnO_2_-CNFi electrodes working under same current rates. Furthermore, when the current rate was reduced to 100 mA g^−1^, the discharge capacity recovered to 274.24 mAh g^−1^, which reaches only 70.4% of the initial capacity, less than the 90.4 % of the SnO_2_-CNFi electrode, and the capacity retention was down to 47.4% (184.5 mAh g^−1^) after 20 cycles. In contrast, capacity retention was up to 96% under the same conditions for the SnO_2_-CNFi electrodes. Table 1 shows a summary of the improvement capacities of SnO_2_-CNFi electrodes in comparison with contrast SnO_2_ sample to prove the better electrochemical performance of SnO_2_-CNFi nanocomposite.

**Figure 9:**
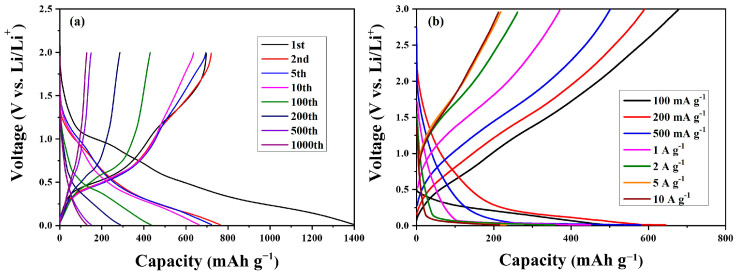
The charge–discharge profiles of the SnO_2_-CNFi nanocomposite at (**a**) 100 mA g^−1^ and at (**b**) various current densities.

Figure 9a,b, respectively, show the typical charge–discharge capacities of the obtained nanocomposite electrode during cycling at a current density of 100 mA g^−1^ and at different current densities in the potential range 0.1–3.0 V (Li/Li^+^). Figure 9a shows the charge–discharge profiles of SnO_2_-CNFi nanocomposite electrode in the 1st, 2nd, 5th, 10th, 100th, 200th, 500th, and 1000th cycles. The discharge capacities of SnO_2_-CNFi at the corresponding cycles are 1397.4, 762.7, 722.3, 662.4, 433.2, 287.5, 148.7, and 128.7 mAh g^−1^, respectively. At the first cycle, the charge and discharge capacities of SnO_2_-CNFi are 696.8, and 1397.4 mAh g^−1^ with a coulombic efficiency is approximately 50%, while the following discharge capacity went down to 762.7 mAh g^−1^. This phenomenon was the result of the formation of a solid–electrolyte interface (SEI) layer on the surface and the decomposition of the electrolyte during the first discharge process, which is also a characteristic phenomenon for metal-oxide nanocomposite anodes. In addition, the carbonaceous matrix can store little lithium but lose initial irreversible capacity, which results in low coulombic efficiency and the decrease in initial discharge capacity [8,9,10,11,12,51,53,54,55]. Furthermore, the decomposition of the electrolyte, the formation of the SEI layer, and the reduction of SnO_2_ to Sn and Li_2_O were confirmed by a plateau identified at around 0.8 V at the first cycle curve [19]. Moreover, the obvious discharge platform observed at approximately 0.8 V during the first cycle disappears in the subsequent charge–discharge curves, and the curve shapes overlap and present similarly, which indicates that the electrochemical stability and cyclability of the SnO_2_-CNFi nanocomposite electrode is moderately and clearly enhanced.

Figure 9b shows the initial discharge–charge profiles of nanocomposite electrodes at different current rates. The initial discharge capacity values of SnO_2_-CNFi nanocomposite were recorded around 679.2, 590.2, 500.6, 370.5, 261.3, 218.2, and 212.1 mAh g^−1^, respectively, at 0.1, 0.2, 0.5, 1, 2, 5, and 10 A g^−1^. At low current densities, the plateaus during the discharge process and during the charging process are not clearly observed, which shows the well-matching results shown in Figure 8a. When higher current rates were applied, the plateau below 0.5 V in the discharge process appeared and was maintained, which evidences that electrochemical redox reactions mainly influence the lithium storage process at high current densities. However, the shapes of these pairs of charge–discharge capacity curves for SnO_2_-CNFi nanocomposite are similar, demonstrating the structural integrity of the electrode and the conversion reactions of transition metal oxide-based electrode are favorably maintained at diverse current densities. In addition, the specific capacities go down gradually as the current densities increase.

Table 2 shows a summary of remaining capacities after cycling to compare the performance between the SnO_2_/carbon material nanocomposites in this work and reported works before. From the table data, it can be seen that there are various types of the carbon materials (graphene, carbon fibers, carbon nanotube) that have been used to prepare the SnO_2_/carbon nanocomposite for high-performance LIBs. However, there are not too many reports using CNFi as an ideal carbonaceous matrix for constructing embedded SnO_2_ high-performance materials. There is a fact that our synthesized nanocomposite exhibited a higher capacity and better cycle performance than other works although some previous work results display better specific capacities than our works for the first 100 cycles. On the other hand, using environmental-friendly and low-cost CNFi from renewable resources could be a significant advantage of our research in the next-generation LIBs industry.

**Figure 10:**
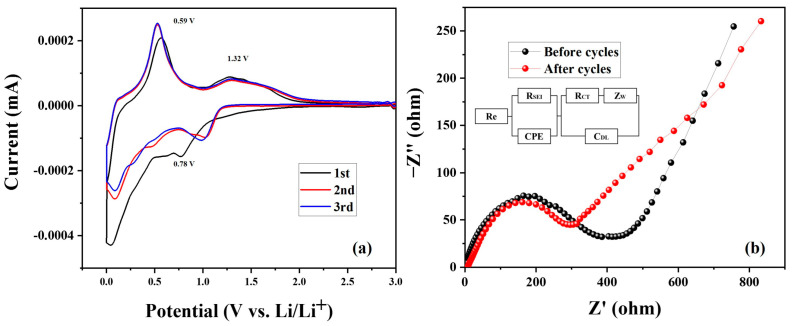
(**a**) Cyclic voltammograms (CV) curves and (**b**) electrochemical impedance spectra (EIS) of SnO2-CNFi electrode before and after 50 cycles.

The cyclic voltammetry curves of SnO_2_-CNFi nanocomposite at a scan rate of 0.1 mV s^−1^ between voltage range 0.01–3.0 V were shown in Figure 10a. During the first cycle, a reduction peak could be observed at 0.78 V and disappear in the following two cycles. This appeared peak is the result of the formation of SEI film during the lithiation process reduced SnO_2_ to Sn (SnO_2_ + 4Li^+^ + 4e^-^ → Sn + 2Li_2_O), which led to the large loss of capacity in the first cycle [13,51,56]. Meanwhile, this peak again confirms the plateau identified at around 0.8 V at the first cycle curve in the charge–discharge tests (Figure 9a). Moreover, two oxidation peaks were obtained at 0.59 and 1.32 V in the delithiation process. The first shape peak at 0.59 V corresponds to the de-alloying process of Li_x_Sn (Sn + xLi^+^ + xe^-^ ↔ Li_x_Sn (0 ≤ x ≤ 4.4)). Meanwhile, the broader oxidation peak at 1.32 V could be explained by the reversible reaction of Sn to SnO_2_ [13,51,56,57,58]. Note that after the first cycle, the curve shape trend is similar, almost overlapping during the delithiation process, and peak intensity becomes higher in the second and third cycle. This phenomenon suggests the SnO2-CNFi nanocomposite has good cycling stability, and these results were in good agreement with the cycling performance (Figure 10a).

Figure 10b showed the comparison of the electrochemical impedance spectra (EIS) of the SnO2-CNFi electrode before and after 50 cycles and the insert verified the circuit model with the symbols as R_CT_, Z_W_, C_DL_, R_SEI_, C_PE_, and Re corresponding to charge–transfer resistance, Warburg impedance, interfacial double-layer capacitance, SEI layer resistance, constant phase element, and electrolyte resistance, respectively. The Nyquist plots consist of a compressed semicircle in the high-frequency region and an increased line in the low-frequency region. The semicircle curve in the high frequency of electrode after 50 cycles had a smaller diameter and the R_CT_ of the electrode showed a decrease from 334.31 Ω to 225.7 Ω after 50 cycles, which demonstrated the improvement of electrochemical performance of SnO2-CNFi and these results could be the reason to explain for the increase in capacity during cycling (Figure 8a).

**Figure 11:**
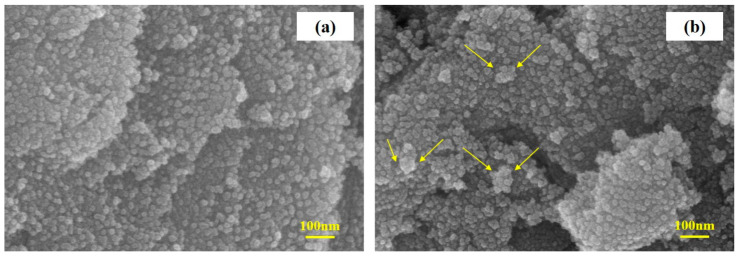
SEM images of the electrode (**a**) before and (**b**) after 200 cycles.

Finally, SEM images of the electrode after 200 cycles were observed to investigate the stability of the nanocomposite stability as shown in Figure 11. The results indicated that the electrode maintained good stability with the formation of SEI film on the surface of the nanocomposite. Except for some small aggregation appears, there was no noticeable change in the nanostructure after 200 cycles. This good stability in the nanocomposite structure could make a significant contribution to the better electrochemical properties and stable cycle performance of the SnO_2_-CNFi nanocomposite electrode.

## 4. Conclusions

In summary, we report on a nanocomposite synthesized by thermally embedding SnO_2_ nanoparticles in cellulose nanofiber. The observed results confirm the successful fabrication of nanocomposite material with the appearance of SnO_2_ nanoparticles and the structure of carbon materials in the final product, greatly improving the performance and preventing the aggregation and volume expansion of SnO_2_. Moreover, the amorphous carbon structure also enhances the stability of SnO_2_ nanoparticles during the charge–discharge process. When utilized in lithium-ion batteries, the nanocomposite electrode could achieve a high specific capacity of 619 mAh g^−1^ at the current rate of 100 mA g^−1^ after 200 working cycles. Especially, the ability to restore and tend to increase the capacity of nanocomposite electrodes after working at high current densities also is a remarkable point for research and development of LIBs electrode materials, working at high current densities.

## Figures and Tables

**Table 1:** Cycling performance capacities of SnO_2_-CNFi/SnO_2_ in comparison at 100 mA g^−1^.

Composite	Initial Capacity	Initial Coulombic Efficiency	Cycle Number	Remaining Capacity	Coulombic Efficiency
SnO_2_-CNFi	1367.6 mAh g^−1^	50.8%	200	619 mAh g^−1^	99.84%
SnO_2_	772.6 mAh g^−1^	49.72%	200	158 mAh g^−1^	99.29%

**Table 2:** Specific capacity summary of previous reports of SnO_2_/carbon materials electrodes.

No.	Composite	Remaining Capacity	Working Cycle	Current Density	Refs.
1	SnO_2_-CNFi (Cellulose nanofibers)	619 mAh g^−1^	200	100 mA g^−1^	Our work
2	am-SnO_2_@p-NC-50%	841.5 mAh g^−1^	100	100 mA g^−1^	[13]
3	Nanofibrous carbon @SnO_2_@MoO_2_ (Cellulose substance)	608.1 mAh g^−1^	100	100 mA g^−1^	[19]
4	CNC-SnO_2_NF800 (nanocrystalline cellulose)	267 mAh g^−1^	500	100 mA g^−1^	[20]
5	SnO_2_/rGO (Graphen oxide)	420 mAh g^−1^	100	395 mA g^−1^	[22]
6	SnO_2_–Fe_2_O_3_/rGO (Graphen oxide)	958 mAh g^−1^	100	395 mA g^−1^	[22]
7	CNF@SnO_2_ (Carbon nanofibers)	469 mAh g^−1^	100	100 mA g^−1^	[23]
8	PCNF@SnO_2_ (Porous carbon nanofibers)	554 mAh g^−1^	100	100 mA g^−1^	[23]
9	GSCN (Graphene-SnO_2_-carbon nanofiber)	1108.1 mAh g^−1^	120	200 mA g^−1^	[24]
10	SnO_2_/Graphene	1156 mAh g^−1^	100	100 mA g^−1^	[51]
11	SnO_2_@carbon core−shell nanocolloids	440 mAh g^−1^	100	300 mA g^−1^	[53]
12	SnO_2_@RHC (rice husk cellulose)	587 mAh g^−1^	100	177 mA g^−1^	[56]
13	Sn/SnO_2_@C-S (Carbon nanofibers)	275 mAh g^−1^	500	500 mA g^−1^	[57]
14	MWCNTs (SnO_2_/Carbon nanotube)	770.6 mAh g^−1^	100	100 mA g^−1^	[58]

## Data Availability

Not applicable.
